# Disseminated Methicillin-Resistant Staphylococcus aureus (MRSA) Infection Masquerading as Community-Acquired Meningitis

**DOI:** 10.7759/cureus.96581

**Published:** 2025-11-11

**Authors:** Mohammed O Hamid, Hashim Ba Wazir, Fatma Salem Alhashemi Alsadi, Jagdeep S Shekhawat

**Affiliations:** 1 Medicine, Sultan Qaboos Hospital, Ministry of Health, Salalah, OMN; 2 Infectious Disease, Medicine, Sultan Qaboos Hospital, Ministry of Health, Salalah, OMN; 3 Radiology, Sultan Qaboos Hospital, Ministry of Health, Salalah, OMN

**Keywords:** linezolid, meningoencephalitis, mrsa, spondylodiscitis, vancomycin

## Abstract

Methicillin-resistant Staphylococcus aureus(MRSA) causes a wide spectrum of infections ranging from simple cutaneous infections to serious invasive infections, including endocarditis and central nervous system (CNS) infections. Multi-site involvement is not infrequently encountered with MRSA infections. Herein, we present a case of complicated MRSA bacteremia presenting as community-onset meningoencephalitis.

A 45-year-old man presented to the emergency department with fever, altered sensorium, and disorientation after receiving diclofenac intramuscular injections. He was diagnosed with meningitis due to MRSA. He was treated with vancomycin and rifampicin. He reported significant low back pain, and lumbaosacral spine MRI showed spondylodiscitis. Moreover, the patient developed right shoulder pain and reduction of movement; a shoulder ultrasound revealed mild joint effusion. As he continued to spike and developed the shoulder pain while on vancomycin/rifampicin, IV linezolid was commenced. As a result, the patient's condition improved and was discharged on oral linezolid followed by cotrimoxazole.

## Introduction

Methicillin-resistant Staphylococcus aureus (MRSA) is a significant pathogen causing a wide variety of community-acquired and nosocomial invasive infections including skin and soft tissue infections, osteoarticular infections, and endocarditis. Central nervous system infections caused by MRSA are often seen in the context of post-neurosurgical interventions in the form of meningitis or ventriculitis.

Community-acquired/spontaneous meningitis is predominantly caused by *Streptococcus pneumoniae* and *Neisseria meningitidi*s, which account for more than 80% of the cases [1]. *Staphylococcus aureus* (including MRSA) is a rare cause of community-acquired meningitis. Several epidemiological studies reported that it accounted for 1 to 9% of bacterial meningitis [2].

The presentation of MRSA meningitis is similar to other causes of bacterial meningitis which includes fever, photophobia and severe headache and neck stiffness [3]. Cerebrospinal fluid findings are consistent with pyogenic meningitis including pleocytosis with predominance of polymorphonuclear cells, reduced glucose level, and raised protein level; thus culture with or without molecular testing is essential for confirming the diagnosis [4].

Vancomycin remains the first-line antibiotic for treatment of MRSA meningitis though it has limitations related to poor CSF penetration, need for therapeutic drug monitoring and toxicity risk [5,6]. Additionally, adjunctive rifampicin is suggested in severe cases when the isolate is susceptible but the evidence for its use is limited. Moreover, therapeutic drug monitoring is recommended to ensure that therapeutic serum concentrations (15-20 mg/L) of vancomycin are achieved [5].

## Case presentation

A 45-year-old man presented to the emergency room with a one-day history of altered sensorium, disorientation, and anorexia. The initial history was obtained from his wife. It was difficult to ascertain if he had any headache, photophobia or other symptoms of meningeal irritation due to his confusional state. All these symptoms developed after receiving diclofenac intramuscular injections for lower back pain. Additionally, he had fever and disturbed sleep for three days prior to admission. The lower back started one week earlier, and it was severe enough to make him visit a local clinic for assessment and pain relief. He was prescribed diclofenac intramuscular injections. After taking the injections, he developed pain and swelling in the gluteal area. He denied any preceding trauma prior to onset of low back pain. He denies illicit drug use. There was no significant past medical history apart from hypertension.

General examination revealed an average built man; he was conscious but disoriented with inappropriate speech and he was not following the doctor's commands. His temperature was 38.2 °C, respiratory rate was 22 per minute, blood pressure was 150/90 mmHg, pulse rate was 136 beats per minute, then came down to 94 beats per minute, and oxygen saturation was 98% on room air. There were no meningeal signs. Neurological examination including motor and sensory, cranial nerves and cerebellar examination was unremarkable. No peripheral stigmata of endocarditis were noted. Cardiovascular examination revealed no murmur. There was no evidence of abscess formation at the injection sites in the buttocks. He had tenderness on palpation of lumbar spine but no signs of nerve root or cord compression. He had normal gait (when he was examined later once the confusional state had improved).

His blood tests showed a high white blood cell count of 18.5 x10^3^/uL, with neutrophils of 16.40 x10^3^/uL, lymphocytes of 0.70 x10^3^/uL, a high C-reactive protein of 292 mg/L and a high alanine aminotransferase (ALT) of 141 U/L. Non-contrast CT scan of the head did not reveal any significant abnormalities. ECG was normal. Therefore, the provisional diagnosis was acute confusional state with possible meningoencephalitis. He underwent lumbar puncture, and the CSF analysis revealed protein of 352 mg/dL, glucose of 0.46 mmol and white blood cells of 880 cells/cmm, which were predominantly neutrophils of 74%. CSF gram stain did not reveal any microorganisms and CSF culture was pending. A summary of the laboratory findings is shown in Table 1. 

**Table 1:** Laboratory findings ALT: alanine aminotransferase, CSF: cerebrospinal fluid

Test	Result	Normal range
WBC	18.5 x 10^3 ^/uL	2.2-10
Neutrophils	16..4 x 10^3 ^/uL	01-May
Hemoglobin	13.6 g/dL	11.5-15.5
Platelet count	193 x 10^3^ /uL	150-450
Creatinine in serum	87 umol/L	45-100
C-Reactive Protein	292 mg/L	0-5
ALT	141 U/L	0-40
Total Bilirubin	11.7 umol/L	0-20
Glycated Hemoglobin	6.40%	4.8-6
Serum glucose	12.7 mmol/L	3.4-7.5
CSF protein	352 mg/dL	15-45
CSF Glucose	0.46 mmol/L	
CSF RBC count	8790 cells/cumm	
CSF WBC count	880 cells/cumm	
Neutrophils % in CSF	74%	
Lymphocytes % in CSF	26%	

He was started empirically on ceftriaxone, vancomycin and dexamethasone to cover community-acquired bacterial meningitis based on the 2004 Infectious Diseases Society of America (IDSA) guideline for treatment of community-acquired bacterial meningitis. CSF culture grew MRSA. Moreover, his blood culture came back positive for MRSA. Dexamethasone and ceftriaxone were stopped based on the culture results. Vancomycin was continued with therapeutic drug monitoring targeting levels between 15-20. In addition, rifampicin was added for adjunctive therapy for MRSA meningitis.

**Figure 1:**
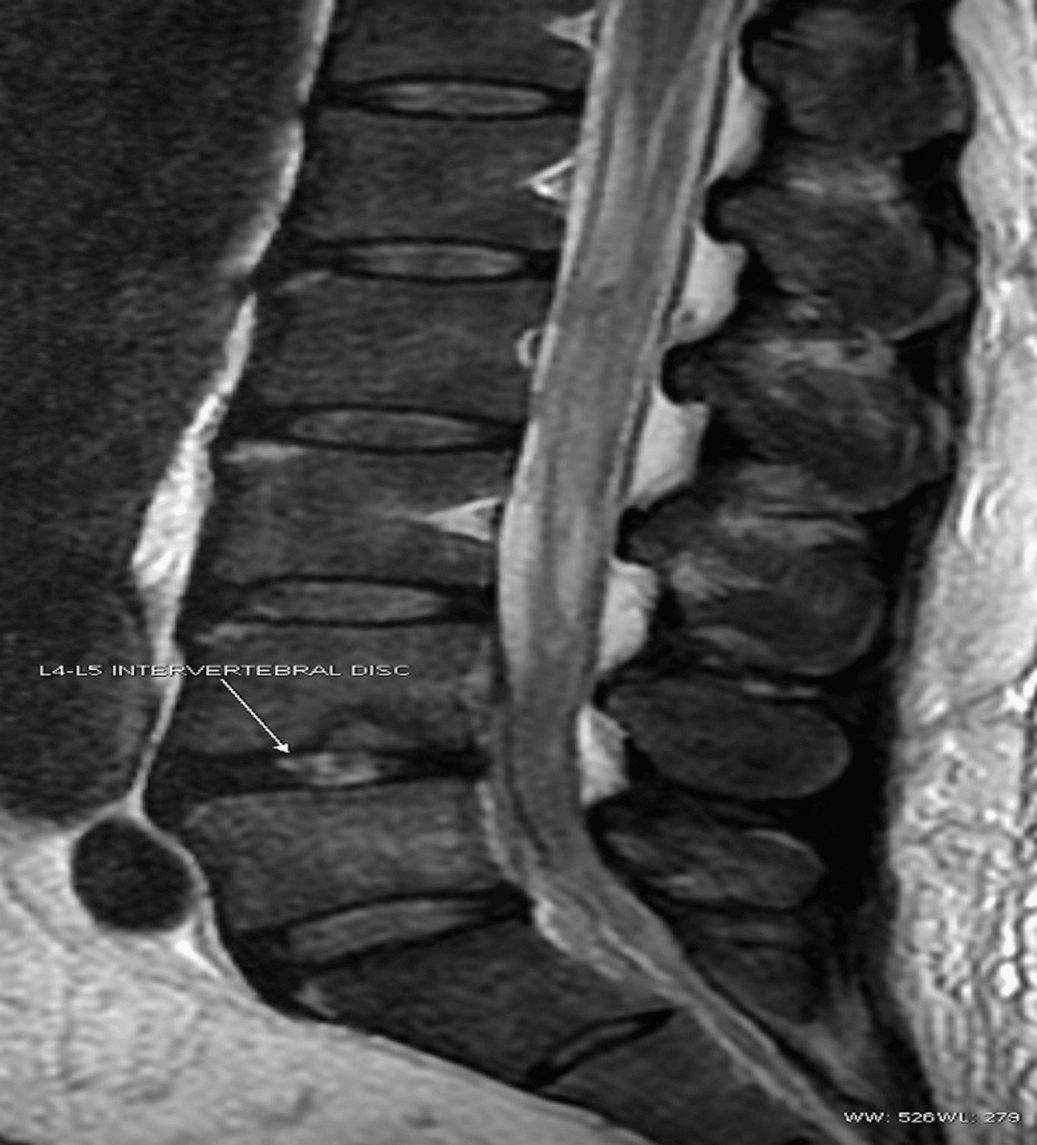
Sagittal T2, short tau inversion recovery (STIR) image.

**Figure 2:**
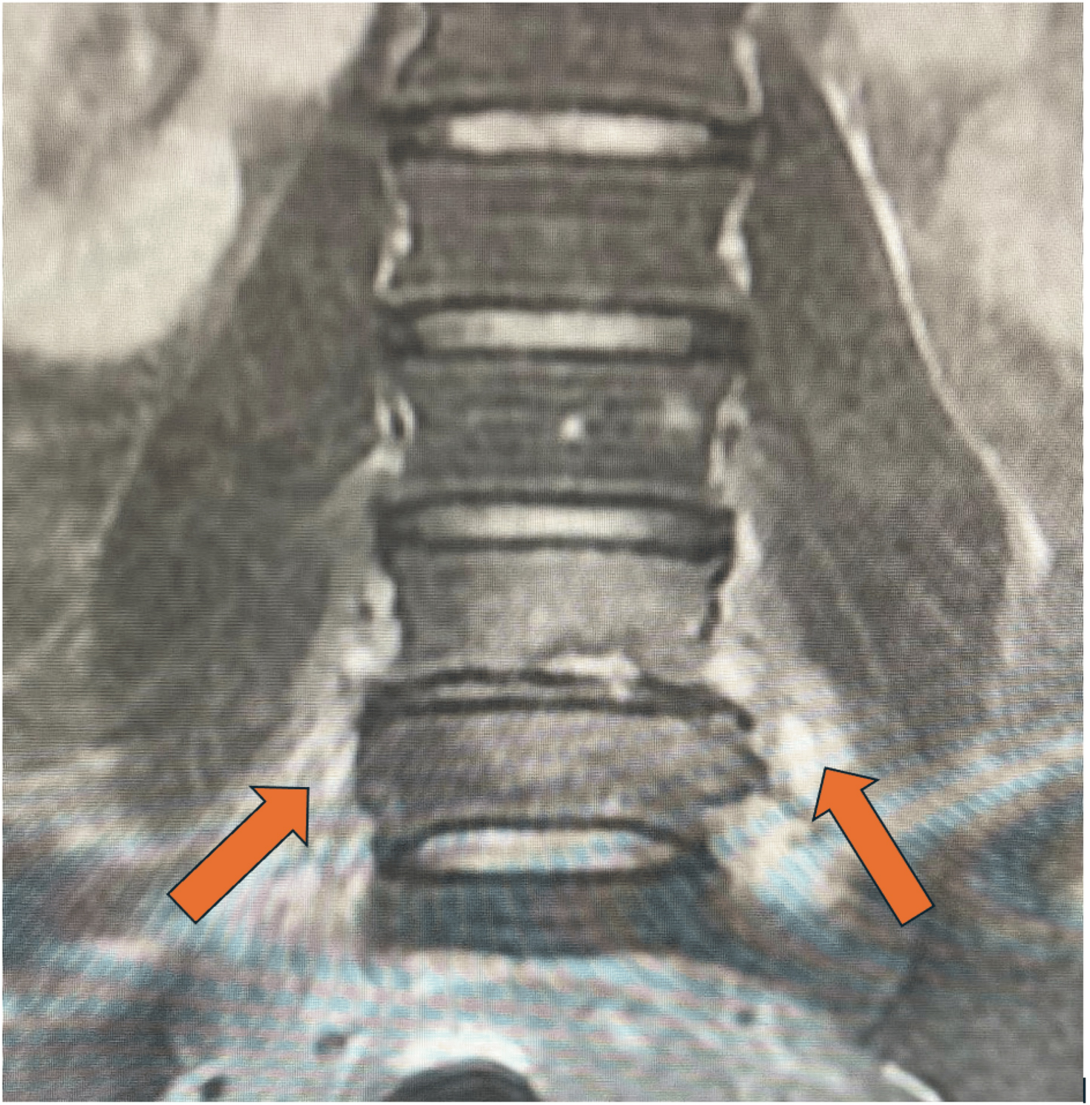
Coronal short tau inversion recovery (STIR) image, signal changes in bilateral paraspinal regions and L4-L5 disco-vertebral region.

Despite vancomycin and rifampicin, the patient continued to have fever, back pain and developed right shoulder pain with restriction of range of movement in the fourth day of admission. Lumbosacral spine MRI demonstrated L5-S1 spondylodiscitis (Figures 1, 2). The shoulder ultrasound revealed a collection of minimal fluid in the shoulder joint. IV linezolid 600 mg twice daily (BID) was added on the fifth day of admission as the fever persisted. Following that, the patient's condition improved, fever subsided and back and shoulder pain were relieved.

Two sets of follow-up blood cultures after starting anti-MRSA therapy were collected (sent on third and sixth days of admission) and reported negative, thus documenting the clearance of bacteremia. CSF culture was not repeated. Transthoracic echocardiogram did not show any vegetation. Trans-esophageal echocardiogram was suggested but after discussion with the cardiologist, a repeat transthoracic echocardiogram was done 10 days following the first transthoracic echocardiogram which again did not show any evidence of endocarditis.

Rifampicin was stopped after 10 days as it was an adjunctive therapy and the patient showed marked clinical improvement. Vancomycin was continued for two weeks, in addition to IV linezolid, then the patient was discharged on oral linezolid 600 mg BID alone for two weeks. He tolerated linezolid well without any evidence of toxicity. Following that, he was switched to co-trimoxazole 960 mg BID for an additional two weeks to complete a total of six weeks duration of anti-MRSA treatment for complicated MRSA bacteremia with spondylodiscitis as recommended by the 2011 IDSA guideline for treatment of MRSA infections.

## Discussion

In this case, the initial presentation of the patient was concerning for meningoencephalitis and the patient was worked up and managed accordingly with lumbar puncture and empiric antimicrobial coverage for community-acquired meningitis. The result of CSF culture was unexpected. Subsequently, the case unfolded to disseminated MRSA infection involving CNS, lumbar spine and possibly the right shoulder. The port of entry for MRSA was probably the intramuscular (IM) diclofenac injections. Although IM injection is a relatively safe and minor procedure, it can rarely lead to serious complications as seen in this case. Most injection site infections are caused by Staphylococcus aureus including MRSA [7]. Skin and soft tissue infections are among the most common primary sources of MRSA bacteremia. Subsequent dissemination to various body sites is not uncommon with MRSA bacteremia, particularly to sites with preexisting degenerative processes or foreign bodies.

There is a paucity of clinical evidence to guide the treatment of MRSA meningitis. The updated UK guideline for treatment of MRSA infections recommends vancomycin and suggests the addition of rifampicin in severe cases though there is no robust evidence to support this recommendation [5]. Similar recommendation is provided in the IDSA MRSA treatment guideline. Linezolid or trimethoprim/sulfamethoxazole are listed as alternative regimens [6].

There is evolving evidence regarding the use of combination therapy for MRSA bacteremia. Different combination regimens have been evaluated with daptomycin and ceftaroline appearing as a promising combination regimen [8]. The addition of a protein synthesis inhibitor like linezolid is suggested in cases of severe sepsis or suspected toxin-mediated processes like necrotizing pneumonia [6].

There has been a paradigm shift in treatment of bacteremia and bone and joint infections from prolonged IV therapy to early switch to oral antibiotic therapy. This is supported by several randomized clinical trials [9,10]. There are several advantages of oral switch, including reduction in the cost (from prolonged hospitalization) and reduced risk of IV-related complications. 

## Conclusions

We present a case of disseminated MRSA infection presenting as community-acquired meningoencephalitis. Clinical vigilance is crucial for identifying both primary and metastatic foci in MRSA bacteremia to facilitate effective source control. This approach ensures the selection of appropriate antimicrobial therapy and optimal treatment duration to maximize therapeutic success.
